# The Modification Mechanism, Evaluation Method, and Construction Technology of Direct-to-Plant SBS Modifiers in Asphalt Mixture: A Review

**DOI:** 10.3390/polym15132768

**Published:** 2023-06-21

**Authors:** Xiang Yan, Di Wu, Kui Hu, Wengang Zhang, Jianbao Xing, Lilong Cui, Silin Shi, Jixu Yang, Chengxu Yang

**Affiliations:** 1 School of Transportation and Vehicle Engineering, Shandong University of Technology, Zibo 255000, China; yxsdlg01@163.com (X.Y.);; 2 Haiwei Engineering Construction Co., Ltd. of FHEC of CCCC, Beijing 101119, China; 3 College of Civil Engineering, Henan University of Technology, Zhengzhou 450001, China; 4 School of Civil Engineering and Geomatics, Shandong University of Technology, Zibo 255000, China; 5 Key Laboratory of Transport Industry of Road Structure and Material, Research Institute of Highway, Ministry of Transport, Beijing 100088, China

**Keywords:** direct-to-plant SBS, asphalt, modifier, material composition, evaluation method, construction technology

## Abstract

To further promote the development of research on direct-to-plant SBS-modified asphalt, this article analyzes the development of direct-to-plant SBS modifiers. Starting from the material composition and mechanism of action, common direct-to-plant SBS modifiers were analyzed and classified into four categories based on their mechanism of action, including the instant dissolution principle, intramolecular lubrication principle, non-granulation principle, and vulcanization principle. From the evaluation of the modification effect, the method of studying the performance of direct-to-plant SBS-modified asphalt is summarized, including fluorescence microscopy, AFM technology, and molecular dynamics simulation technology. From the perspective of practical application, the construction process of direct-to-plant SBS-modified asphalt was discussed, including the design stage, raw material preparation stage, mix design stage, and on-site construction stage. The results show that common direct-to-plant SBS modifiers are primarily SBS with a small particle size (less than 200 mesh) or specific model, supplemented by additives (EVA, naphthenic oil, sulfur, petroleum resin, etc.), which improve melting efficiency and lubricity or make it undergo vulcanization reaction, change the proportion of asphalt components, and improve stability. In the evaluation of the modification effect of direct-to-plant SBS-modified asphalt, the disparity of the direct-to-plant SBS modifier is determined by observing the particle residue after dry mixing. Macroscopic indexes of modified asphalt and modified asphalt mixture are used to determine the cross-linking effect of direct-to-plant SBS modifier and asphalt, and the modification mechanism and modification effect of wet SBS modifier are evaluated at the microscopic level. The development of direct-to-plant SBS-modified asphalt should combine the characteristics of direct-to-plant SBS modifiers and the attributes of field application, targeted research, and the development of high-performance direct-to-plant SBS modifiers and complete production technologies applicable to different regions, strengthen the improvement of modification effect evaluation, and form a complete theoretical system.

## 1. Introduction

SBS is a triblock copolymer composed of styrene and butadiene as monomers and is the world′s largest thermoplastic elastomer with the most similar properties to rubber. SBS-modified asphalt is the most widely used type of modified asphalt in the world, and its research is also relatively extensive and in-depth. SBS can improve the rutting resistance of asphalt mixture [1,2] and enhance low-temperature cracking resistance [3,4], fatigue cracking resistance [5,6,7], and water damage resistance [8,9]. Therefore, this kind of asphalt is also used in the highest proportion in highway engineering construction and occupies an absolute advantage in highway application. The traditional production process of SBS-modified asphalt is the wet modification process, which is also the mainstream production process today. The wet modification process is to transport the base asphalt and modifier to the modified asphalt plant, and after mixing in a particular proportion, the SBS modifier is ground and dispersed by a significant shear or colloid mill and fully swelled to produce finished SBS-modified asphalt. After being transported to the construction site, the mineral powder is added, and the aggregate is mechanically mixed to make an SBS-modified asphalt mixture for construction. The practice has proved that wet-modified asphalt has many disadvantages. In addition to high energy consumption, wet SBS-modified asphalt still has two problems that seriously affect its technical performance and have not been overcome up to now. One is the separation of the SBS modifier during high-temperature storage and transportation, and the second is the fracture and degradation of SBS molecules at high temperatures during processing. After the SBS-modified asphalt is processed, during transport and paving, affected by time and temperature, the softening point and ductility will continue to decay [10,11]. The construction process is becoming richer and richer with the increasing variety of modifiers. Direct-to-plant SBS-modified asphalt, also known as dry-process SBS-modified asphalt, was put forward as a road material. In terms of chemical composition, the wet SBS modifier is a styrene butadiene styrene triblock copolymer. The direct-to-plant SBS modifier is primarily composed of crushed SBS, supplemented by additives. The types of additives vary depending on different types of direct-to-plant SBS modifiers, including EVA, naphthenic oil, sulfur, petroleum resin, etc. When making direct-to-plant SBS modifiers, SBS and additives are mixed evenly in a certain proportion, and then they are fed into a twin-screw extruder for melting and granulation (particle size less than 2 mm) to obtain the finished direct-to-plant SBS modifier. The direct-to-plant SBS modifier is directly put into the mixing plant. It is rapidly fused with the aggregate and base asphalt in a short-term mixing process to achieve micron-level dispersion and form a stable cross-linked structure with the asphalt to achieve the purpose of modification. Compared with the wet modification process, the direct-to-plant modifier avoids the problem of quality control in the production process of the asphalt mixture, and there is no need for special production equipment, long-term high-temperature production storage, and transportation. It saves the production of modified asphalt in the factory, so it has the advantages of low cost, low energy consumption, low emissions, no segregation, and a simple and fast process.

There are more research techniques for direct-to-plant of SBS-modified asphalt. However, most need to be expanded to the laboratory, are not industrialized, or are only used in a small area. In the direct-to-plant SBS modifier material composition and mechanism of action, direct-to-plant SBS-modified asphalt modification effect evaluation, and construction process, many problems remain to be solved. Regarding direct-to-plant SBS modifier research and development, there is yet to be a high-performance direct-to-plant SBS modifier effect evaluation, there is a lack of targeted evaluation methods, and the construction process is still the traditional SBS-modified asphalt mix process for construction. On the premise of the above problems, this paper systematically compares the existing research on direct-to-plant SBS-modified asphalt, comprehensively analyzes the material composition of different direct-to-plant SBS modifiers, the mechanism of action, and the current problems, summarizes the research methods for evaluating the modification effect of direct-to-plant SBS-modified asphalt, and elaborates on a detailed construction process. The research results of this paper aim to provide a reference for the subsequent research of direct-to-plant SBS-modified asphalt.

**Figure 1:**
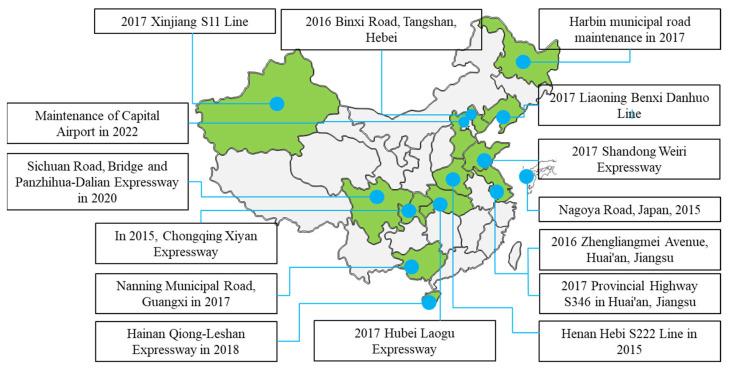
Distribution diagram of dry SBS-T modifier test road.

Research on direct-to-plant modifiers for asphalt modification was initiated worldwide in the 19th century. In France, RP modifiers were developed to significantly improve the rutting resistance of asphalt pavements. In 1992, specifications for producing and constructing direct-to-plant high-modulus asphalt mixtures were developed [11,12]. In Japan, TPS direct-to-plant modifiers suitable for drainage pavements were developed, which can significantly improve the strength and durability of asphalt mixtures [13]. Germany developed a direct-to-plant Duroflex modifier, which can dramatically improve the low-temperature crack resistance of asphalt mixtures and the self-healing ability of pavement cracks [14]. Long-life pavements were introduced in the United States using direct-to-plant high-modulus asphalt mixtures as the middle and lower layers [15]. Greenland used a dry process to incorporate SBS-based modifiers into modified asphalt mixtures, which are more resistant to rutting and more adaptable to arctic weather than conventional ones [16]. China Beijing Lu You Materials Co., Ltd (Beijing, China). produces direct-to-plant high-viscosity modifiers for modified asphalt with low viscosity and high viscosity at low temperatures, good construction, and ease. The SBS-T modifier produced by China Road High-tech (Beijing, China) Engineering Technology Research Institute Co, Ltd. can melt quickly and improve modification efficiency. Direct-to-plant modifiers can be uniformly dispersed and quickly melted in asphalt and have significant energy-saving effects compared to traditional wet mixing processes. The mechanism of action is divided into three sub-processes: Melting, viscous flow, and melt fusion [17,18]. In recent years, the microstructure and macroscopic properties of direct-to-plant SBS-modified asphalt have been studied more and more intensively. Yu used a fluorescence microscope to compare and analyze the performance of wet- and dry-modified asphalt and introduced the rapid modification mechanism analysis and modification effect evaluation of direct-to-plant SBS modifier [19]. Direct-to-plant SBS-modified bitumen experimental roads are also currently being paved in many provinces in China (as shown in Figure 1). The Technical Guide for Highway Dry SBS-Modified Asphalt Pavements (T/CHTS 20003-2018) was also promulgated immediately afterward, which provides detailed provisions specifying the technical specifications of direct-to-plant SBS modifiers, as well as the design methods, mixing processes, and construction techniques for direct-to-plant SBS-modified asphalt mixtures, and is the only technical guide/standard to date involving direct-to-plant SBS modifiers [20].

## 2. Material Composition and Action Mechanism of Direct-to-Plant Modified

### 2.1. Direct-to-Plants Modifier Based on the Instant Dissolution Principle

This type of direct-to-plant modifier is based on star-shaped SBS, which can reduce the particle size of SBS through crushing, speed up the reaction rate, assist with ethylene-vinyl acetate copolymer (EVA), naphthenic oil, etc., and use a double-screw rod machine for granulation. Upon being directly placed into the mixture during mixing, such direct-to-plant-modifiers can rapidly melt, cause swelling, change the original structure, and significantly improve the comprehensive performance of the mix [21].

#### 2.1.1. Material composition

There has been comprehensive domestic and foreign research and engineering practice in terms of material design. The primary raw material of the direct-to-plant SBS modifier based on the principle of quick dissolution is an SBS modifier with a particle size of less than 200 mesh, supplemented by an ethylene vinyl acetate copolymer (EVA), naphthenic oil, a compatibilizer, antioxidant, etc., which is made by mixing, melting, granulation, and cutting [21,22]. EVA/SBS and naphthenic oil/SBS are the critical component parameters affecting direct-to-plant SBS modifiers. In contrast, an optimum aromatic oil/SBS allows direct-to-plant SBS to have the best swelling rate and swelling effect [23,24].

#### 2.1.2. Mechanism

**Figure 2:**
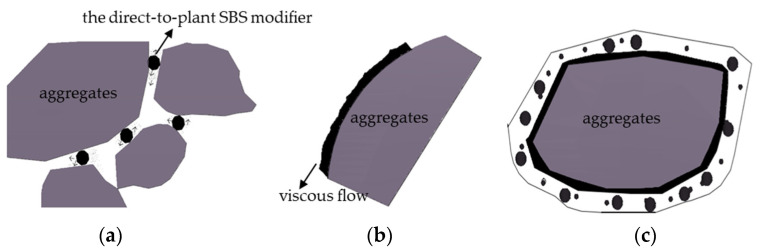
The mechanism of direct injection SBS modifier [18], (**a**) represents a mixed solution stage, (**b**) represents a viscous flow stage, and (**c**) represents a swelling crosslinking stage.

The modification process for direct-to-plant SBS modifiers based on the instant dissolution principle also involves mixing, dissolving, swelling, and cross-linking. During the dry mixing process of the modifier with the aggregate, the high temperature of the aggregate is transmitted to the modifier particles, which first causes the particles to melt and enter a viscous flow state from a high elastic state. Under the combined action of compressive shear force, stirring centrifugal force, and gravity of the aggregate, the melt undergoes viscous flow, and the modifier particles form a micrometer-scale film on the surface of the aggregate. When sprayed into asphalt, the asphalt encapsulates the modifier solution, and the modifier molecules diffuse toward the asphalt binder. Promoted by the strong shear of the aggregate, a mechanical blend of the asphalt solution and the modifier solution is formed quickly, forming a uniformly modified asphalt mixture. As shown in Figure 2.

**Figure 3:**
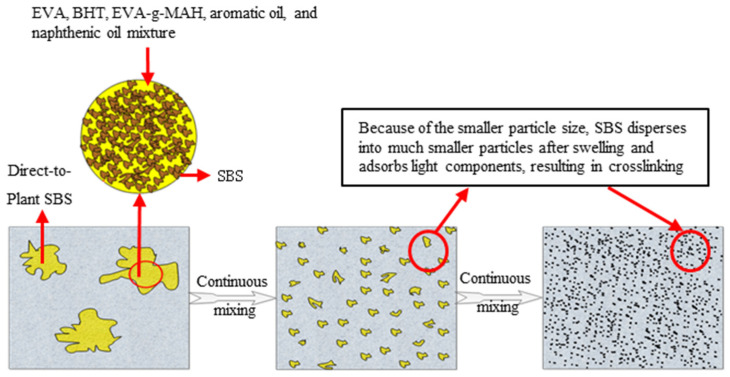
Schematic diagram of dispersion and swelling process direct-to-plants modifier in asphalt [24].

The direct-to-plant SBS modifier based on the instant dissolution principle can quickly complete the mixing, dissolving, swelling, and cross-linking process because it contains EVA components. The solubility parameters of asphalt are very close, which can improve the weakness of SBS and asphalt compatibility. At the same time, compatibilizers can introduce strong polar reactive groups to improve the polarity and reactivity of the material, causing SBS and EVA to have a robust interfacial interaction, which in turn enhances the compatibility of the two substances [21,22,23,25]. As the ratio of EVA/SBS increases, the adsorption and swelling of the direct-to-plant SBS modifier in the asphalt are improved, and the properties of the resulting modified asphalt mixes are all enhanced [24]. The solubility parameters of SBS and asphalt fluctuate with the change in temperature. SBS has a significant impact on the molecular structure of asphalt. After SBS is added to asphalt, due to the small particle size and high surface energy, it can adsorb the light components of asphalt, swell, change the spatial structure of asphalt, improve the original compactness of asphalt, and greatly enhance the activity of branch chains of asphalt components [26,27]. The mechanism of modification of asphalt by a direct-to-plant SBS modifier is not only the general physical effects of mixing, dissolving, and swelling but also the formation of irreversible chemical bonds through cross-linking under certain conditions, thus forming a stable three-dimensional network structure, achieving the same effect as a wet modification. The SBS particles split into smaller particles when they absorb the lighter components of the asphalt and swell and cross-link (as in Figure 3) to give the asphalt better elasticity and higher strength, improving its road properties, including its fatigue resistance, aging resistance, and high and low-temperature performance [28,29].

### 2.2. Direct-to-Plants Modifier Based on Intramolecular Lubrication Principle

SBS is an excellent performance asphalt modifier and is the most widely used worldwide [30,31]. Its considerable molecular weight, ranging from tens to hundreds of thousands, will increase the difficulty of dissolving SBS in asphalt mixtures. When melted into a liquid state by heat, the flow and dispersion performance under melting liquid form could be better due to its disordered and entangled molecules, which increases intermolecular friction, so conventional SBS has poor dissolution performance with common petroleum asphalt [32,33,34]. Modern SBS-modified bitumen production goes through “dissolution, shear milling, development, stabilization” and other production processes to produce SBS-modified bitumen to meet construction requirements. Otherwise, due to the lack of SBS liquidity and incomplete reaction, the asphalt mixture will not meet the standard. These direct-to-plant SBS improvers use the principle of intramolecular lubrication. The SBS molecules are combined with petroleum resin molecules and lubricant molecules using a double-screw rod extruder. When producing SBS-modified bitumen in a dry process, it is directly injected. It can be dispersed quickly in the aggregate and matrix asphalt, formulating a direct-to-plant SBS-modified bitumen mix with excellent performance and ease of use.

#### 2.2.1. Material Composition

Material design: The primary raw materials for the direct-to-plant SBS modifier based on the principle of intramolecular lubrication are 4303-star SBS and 1301 linear SBS in a 1:1 ratio, supplemented by petroleum resins, lubricating oils, and calcium carbonate. It is sheared, extruded, dispersed, and kneaded by a double-screw rod machine [35]. Different types of SBS have other modification effects on asphalt. Star SBS is more effective than linear SBS at improving the high-temperature stability, low-temperature crack resistance, and water stability of SBS-modified asphalt mixes. Choosing the appropriate structure type when modifying asphalt is crucial [36,37,38]. Aided by the lubricating oil (which reduces friction), the petroleum resin molecules benefit from their excellent mobility at high temperatures, interspersing into the network of SBS molecules and improving the dispersion of SBS molecules.

#### 2.2.2. Mechanism

**Figure 4:**
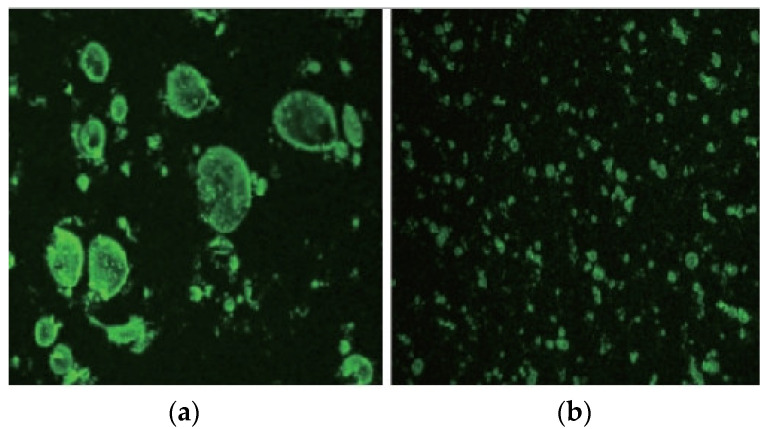
Fluorescence micrograph of SBS-C9 asphalt [41], (**a**) Represents the fluorescence microscopic image of C9 petroleum resin not added, and (**b**) represents the fluorescence microscopic image of C9 petroleum resin added.

These direct-to-plant SBS modifiers use the principle of intramolecular lubrication, where the petroleum resin molecules and the lubricant molecules are small organic compounds with polar groups. These small molecules are evenly distributed between the structural units of the SBS macromolecules, thus making it easier for the SBS molecules to move and not changing the main properties of the copolymer, while also reducing the friction between the molecules and improving the flow and dispersion of the polymer [39,40]. Figure 4(a) shows that without adding the C9 petroleum resin, the SBS modifier’s distribution is more aggregated and forms larger groups. In contrast, the interface between the SBS group and the bitumen is straightforward, indicating that the SBS is relatively aggregated in the bitumen dispersion. Figure 4(b) shows that the SBS distribution is more dispersed after the addition of the C9 petroleum resin, and the aggregation group is smaller, indicating that the compatibility between SBS and asphalt is significantly improved by the compounding effect of the C9 petroleum resin, resulting in a more uniform dispersion of SBS [41].

The SBS molecular structure of each butadiene chain segment end is connected to a styrene block. The two ends of the styrene block are the hard block in SBS, which are easily physically cross-linked together in the region and form a dispersed phase to improve SBS performance [42]. The butadiene chain segments are also polymerized to start a continuous phase, and incompatibilities exist between polystyrene and polybutylene. Through the aggregation of polystyrene, SBS forms a three-dimensional structure, and the end groups of polystyrene contribute to the strength of the bitumen. Still, when melted into a liquid state by heat, the molecular arrangement is more complex, resulting in increased intermolecular friction. By using the excellent fluidity of petroleum resin and lubricating oil at high temperature, the SBS is mixed with the SBS, and after shearing, extruding, dispersing, and kneading in the double-screw extruder, the SBS is incorporated into the petroleum resin and lubricating oil, and the friction between the SBS molecules is reduced. The lubricity is enhanced through the “force-chemical bonding” without lowering the performance of the SBS-modified bitumen.

### 2.3. Direct-to-Plant SBS Modifier Based on the No-Pelletizing Principle

Modern conventional direct-to-plant SBS modifiers all require a double-screw rod extruder for cutting and pelletizing, which first carries out the melting process of the granular modifier during modification, reducing the reaction rate of the direct-to-plant SBS modifier and increasing the melting time of the direct-to-plant SBS modifier. Hence, Tang proposed a direct-to-plant SBS modifier based on the no-pelletizing principle. The small particle size (1–100 μm) of these direct-to-plant SBS modifiers, with small strip burrs on the surface, increases the contact area and accelerates melt dispersion without the need for granulation, making them a new type of direct-to-plant SBS direct-to-plant asphalt modifier that can be put in directly [43].

#### 2.3.1. Material Composition

In terms of material design, these direct-to-plant SBS modifiers are based on SBS with a particle size of 2000 μm or less, supplemented by crosslinking agents (sulfur or sulfur compounds) and crosslinking aids, with burr generators (iron-based alloy powder or nickel-based alloy powder) added to make the SBS surface finely hairy, and abrasives and filling oils added during the granulation process to improve high-temperature fluidity [43].

In this direct-to-plant modifier, low-speed grinding of SBS powder is critical. Using burr-generating agents allows for slippage and misalignment during the grinding of SBS. Because SBS is challenging, the slippage or misalignment will rotate around a point and cannot be broken, thus creating burrs of varying lengths on the surface of SBS particles.

#### 2.3.2. Mechanism

The no-pelletizing direct-to-plant SBS modifier improves production efficiency by increasing the contact area during the reaction. Due to the small particle size of SBS and fine strip-like burrs on the surface, the granulation process is avoided while increasing the contact surface area. This greatly improves production efficiency and solves the problem of modifier floating while not affecting the performance of the mixture. When a modifier containing an appropriate amount of water is added to the mixing plant, under the action of water, the modifier particles will form an association with a certain shape but not tightly, which can counteract the floating phenomenon caused by external force interference. When entering the mixing pot and encountering high-temperature mixing, a small amount of water can quickly evaporate without affecting the modification effect of the modifier on asphalt. Adding filler oil can effectively increase the high-temperature flowability of direct injection modifiers and improve the melt index. When mixed with high-temperature stones in the mixing plant, it promotes the rapid melting of the modifier [43,44,45,46].

### 2.4. Direct-to-Plants Modifier Based on Vulcanization Principle

The solubility of SBS and matrix asphalt is very different. The two are physically mixed and prone to phase separation at high temperatures, seriously affecting the high-temperature stability of the modified asphalt and causing the quality to not meet the requirements during construction [47,48,49,50]. Several studies have been carried out to improve the high-temperature stability of modified bitumen. Different types of base bitumen and other preparation conditions affect the hot storage stability of modified bitumen; specifically, base bitumen containing more aromatic fraction has more desirable storage stability. Modified bitumen prepared by the pre-swelling process of bitumen during preparation can improve storage stability [51]. Adding a certain amount of inorganic clay (diatomaceous earth, montmorillonite) to prepare modified bitumen results in excellent high-temperature strength of the asphalt mixture [52]. Adding carbon nanotubes (CNT) as a binder to the modified bitumen reduces the needle penetration and increases its kinematic viscosity and softening point, enhancing stability [53,54]. In research into improving the stability of asphalt mixtures, scholars have found that by adding sulfur to the modified bitumen, a vulcanization reaction with the asphalt mixture can significantly enhance its stability. During the vulcanization process, sulfur and modified bitumen cross-linking occur, producing large molecules containing sulfur. XPS and potentiometric titration identified the main products (disulfide, sulphoxide) following the vulcanization reaction between sulfur and modified bitumen [55]. By comparing the effects of polymeric sulfur and elemental sulfur on the performance of modified bitumen, Ming [56] concluded that SBS-modified bitumen has higher storage stability when polymeric sulfur with a high sulfur content is used as an admixture. Thus, Yuan [57] proposed a new preparation scheme for direct-to-plant SBS modifiers based on the vulcanization reaction and verified the performance. The direct-to-plant SBS modifier based on the vulcanization reaction changes the asphalt’s components through the vulcanization reaction, significantly improving the modified asphalt′s storage stability while meeting the premise of direct-to-plant construction. Luo [27] developed a direct-to-plant SBS modifier using the cross-linking reaction between SBS, sulfur, and waste bio-oil. The bio-oil/sulfur composite SBS-modified asphalt was modeled by molecular simulation, and the performance was compared with that of wet SBS-modified asphalt. Tests on asphalt mixes were also carried out. The results showed that the composite direct-to-plant-modified asphalt mixes met the standards of wet-modified asphalt mixes regarding high- and low-temperature performance and water stability.

#### 2.4.1. Material Composition

The direct-to-plant SBS modifier based on the Vulcanization principle proposed by Yuan was based on an SBS modifier with a particle size of less than 200 mesh, supplemented by sulfur (S), tetramethyl thiuram disulfide (TMTD), zinc oxide (ZnO), and kaolin (2SiO_2_–Al_2_O_3_–2H_2_O), and the optimum parameter was determined as the ratio of sulfur (S), tetramethyl thiuram disulfide (TMTD), zinc oxide (ZnO), and kaolin (2SiO_2_–Al_2_O_3_–2H_2_O) masses of 60:16:4:1750 [57].

#### 2.4.2. Mechanism

**Figure 5:**
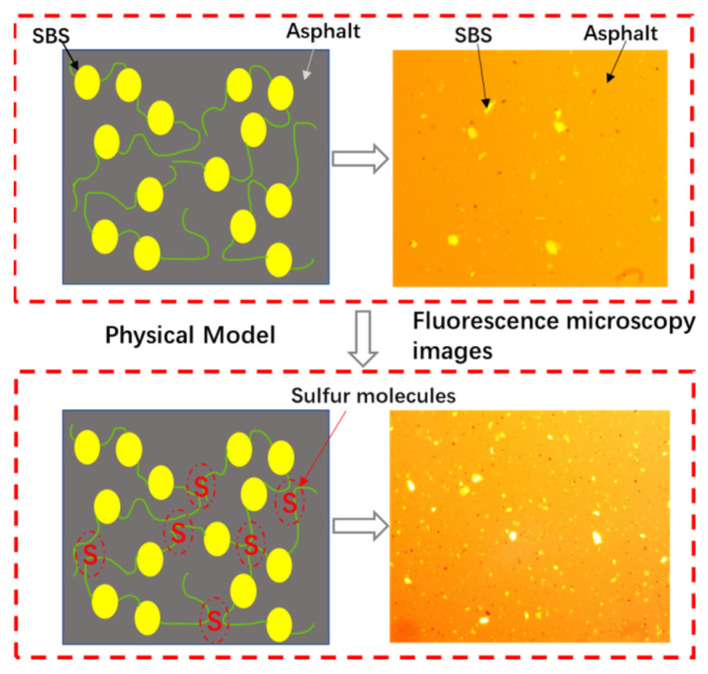
Sulfur crosslinking reaction mechanism.

This type of direct-to-plant SBS modifier primarily improves its thermal storage stability. Sulfur is a high-performance stabilizer, and when sulfur is added to the asphalt mixture, it will undergo a cross-linking reaction. The sulfurization reaction between asphalt and sulfur will occur, causing a change in the proportion of asphalt components. The asphalt sub-chain will change from a two-dimensional chain structure to a three-dimensional network structure, generating sulfur-containing macromolecular components, making the asphalt more stable and increasing viscosity, as shown in Figure 5 [58,59]. When nano-ZnO is used as an auxiliary admixture, it can increase the softening point and consistency of the matrix asphalt and form a stable modified asphalt system with the matrix asphalt, which enhances the high-temperature stability, low-temperature crack resistance, and water stability of SBS-modified asphalt [60,61,62].

## 3. Evaluation Method of Modification Effect

At present, the dispersibility of the direct-to-plant SBS modifier is primarily determined by observing the residual particles after mixing, and the macroscopic indicators of modified bitumen and modified bitumen mixes are used to determine the cross-linking effect of the direct-to-plant SBS modifier with the bitumen. Generally, the technical indicators of asphalt mixtures, such as the residual stability ratio in the immersion Marshall test, the residual strength ratio and dynamic stability in the freeze-thaw splitting test, the bending stiffness modulus in the low-temperature bending test, and dynamic stability in the high-temperature rutting test, are indirectly evaluated using direct-to-plant SBS-modified asphalt mixtures, including their high- and low-temperature rheological properties [63,64,65,66]. Prepare the direct-to-plant SBS-modified asphalt samples using the same method as the wet-modified asphalt preparation process (shear dispersion + developmental swelling), and directly evaluate technical indicators such as penetration, softening point, ductility, viscosity, and shear modulus [67,68,69].

**Figure 6:**
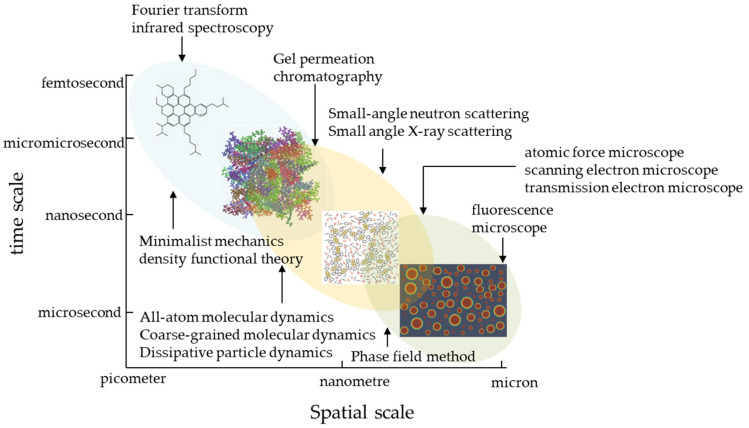
Experimental and simulation methods for the microstructural composition of bitumen [70].

The experimental and simulation methods in the microstructural composition of asphalt are shown in Figure 6 [70]. Analysis of relevant domestic and international data reveals very little information involving the study of the modification effect of direct-to-plant SBS modifiers. Still, there is more literature on the modification mechanism and impact of wet SBS modifiers, which can provide a reference for the modification effect of direct-to-plant modifiers. The existing studies use fluorescence microscopy and atomic force microscopy together with the phase field method to capture and calculate the modifying impact of direct-to-plant SBS modifiers on asphalt during the mixing process of asphalt mixtures and use molecular dynamics simulation to determine the diffusion and adhesion patterns of asphalt components during the swelling process. The current status of related research is summarized below.

### 3.1. Analysis of the Microscopic Morphology of SBS-Modified Bitumen Based on Fluorescence Microscopy

The SBS-modified bitumen can be divided into SBS and bitumen phases using fluorescence microscopy. In recent years, fluorescence microscopy has played a more significant role in detecting SBS content in modified bitumen [71,72]. Some scholars believe that the percentage of SBS modifier particle area and the ratio between the long and short axes are closely related to the modification effect in the fluorescence micrographs [73,74]. Laukkanen et al. expressed the swelling process of SBS by studying the percentage of lighter bitumen components under fluorescence microscopy and observed a continuous SBS-rich network structure [75]. Some scholars combined fractal dimension, infrared spectroscopy, and atomic force microscopy (AFM) with fluorescence microscopy to analyze the distribution and swelling state of SBS in bitumen [76,77,78,79]. Liang et al. used fluorescence microscopy to establish an SBS-modified bitumen phase field model. They interpreted the phase field method for the time-containing evolution of the microscopic phase state of SBS-modified bitumen, making fluorescence microscopy a significant step forward from qualitative to quantitative studies of SBS-modified bitumen [80]. Of course, Tan pointed out that although the phase field method is essential for studying phase behavior in bitumen microstructures, its classification of bitumen components and phase states is still relatively primary [70]. In practice, the phase composition of the internal microstructure of bitumen is strictly divided according to the four parts, and such issues need to be further explored in future research.

**Figure 7:**
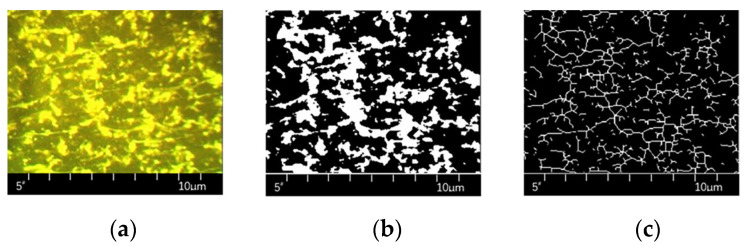
Fluorescence micrographs of direct-to-plant SBS-modified bitumen [24], (**a**) is the fluorescence microscopy RGB image, (**b**) is the binary image, and (**c**) is the skeletonized image [24].

In addition, the acquisition and processing of fluorescent microscopic images of SBS-modified bitumen is the basis for quantitative analysis, of which the most critical step is binarization. Standard binarization thresholding algorithms include OTSU, Iterative, Niblack, NFCM, Bersen, etc., which can be divided into global and local thresholding according to the thresholding method. Various shooting parameters, such as magnification and exposure time during fluorescence image acquisition, can significantly impact image quality [81]. Improvements in binarized thresholding algorithms are an essential way to solve the above problems, such as the smoothing of images, improvements in two thresholding algorithms, the creation of thresholding surfaces, and target segmentation using deep convolutional neural network theory [82,83]. Observing and capturing the direct-to-plant SBS-modified asphalt using a fluorescence microscope, and using MATLAB software to analyze and quantitatively calculate the microstructure of the direct-to-plant SBS modifier, as shown in Figure 7. 

In conclusion, the acquisition of fluorescence microscopy red–green–blue (RGB) maps is currently relatively mature, and it is generally believed that the degree of cross-linking of SBS under the microscope is a direct reflection of its effect on asphalt modification. However, limited by the limitations of cross-discipline, when obtaining the relevant quantitative parameters of the degree of cross-linking, most of the RGB maps of SBS-modified asphalt are binarized using the global threshold method, lacking a targeted binarization threshold segmentation method, resulting in the RGB map information being locally amplified or ignored, which essentially limits the accuracy of quantitative analysis of the degree of dissolution of SBS-modified asphalt using fluorescence microscopy.

### 3.2. Analysis of Asphalt Nano Morphology based on AFM

**Figure 8:**
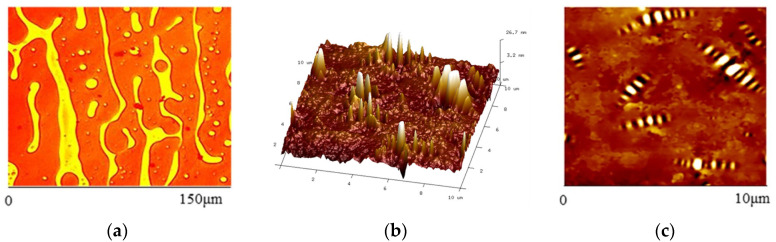
RGB schematic diagram and AFM schematic diagram, (**a**) represents the RGB image, (**b**) represents the AFM three dimensional morphology diagram, and (**c**) represents the AFM two dimensional morphology diagrams.

The AFM technique provides a technical way to study the nano morphological characteristics of the asphalt. The existence of a “bee-like structure” on the surface of bitumen and modified bitumen is generally accepted in the literature, but its formation mechanism is more controversial [84,85,86,87,88,89,90,91]. The current mainstream view is that the presence of ′wax′ is an essential factor affecting the shape of asphalt under AFM [92,93,94,95]. In terms of the location and conditions of the ′bee-like structure, Ramm et al. used non-contact optical microscopy and optical scattering techniques to suggest that the asphalt ′bee-like structure′ is only present on the surface and does not appear immediately during the heating–cooling cycle [96]. De Moraes et al. found that when the bitumen temperature exceeded 57 °C, the “bee-like structure” would disappear [97]. When analyzing AFM diagrams, most scholars have used indicators such as the reduction in the number of “bee-like structures,” the degree of aggregation, surface height, and the reduction in surface roughness [98,99,100,101,102,103,104]. For example, Xing et al. proposed a new AFM sample preparation method based on the hot-cast method [105]. They recommended suitable probe types and parameters for AFM testing, which significantly improved the quality of AFM imaging and the stability of test results. Our research group mixed the direct-cast SBS modifier, asphalt, and aggregates (clean coarse aggregate, no fine aggregate, and mineral powder) at a temperature of 185 °C, absorbed the asphalt, and obtained the RGB image of the direct-cast SBS-modified asphalt using a fluorescence microscope. The corresponding AFM image was also obtained using AFM. The RGB schematic and AFM schematic are shown in Figure 8.

In addition, many scholars have attempted to establish a link between the surface morphological parameters of asphalt AFM and aging performance [100,106,107,108,109,110]. Some scholars have also applied AFM to modified bitumen. Dehouche et al. used AFM to study the degree of homogeneity of organic montmorillonite-modified bitumen [111]. Meng et al. used AFM to investigate the effect of aging regeneration on the binding properties of modified bitumen binders [112]. Li proposed using an atomic force microscope to quantitatively characterize the adhesion between the binder and aggregate mineral particles at the micro-scale and directly measure the adhesion between aggregate minerals and the binder substrate [113]. Comparative tests found that the unit surface energy was the largest in the alumina binder pair and relatively small in the calcium carbonate and silica binder pairs. Ye et al. successfully performed phase structure and quantitative analysis of nano montane/SBS-modified bitumen AFM images [114]. Shan et al. classified SBS-modified bitumen into bee-like and interstitial forms based on AFM images and analyzed the microscopic damage mechanism of the bitumen by combining the AFM test results and finite element simulation results [115].

In conclusion, AFM technology has become essential for studying and modifying bitumen. Some progress has been made in terms of asphalt nano morphological testing methods, the relationship between morphological characteristics and macroscopic mechanical parameters, and the factors influencing changes in morphological traits, but the quantification of morphological features and related properties is still in its infancy, and the quantitative analysis of different phases in the asphalt AFM phase diagram will become a breakthrough point in this field.

### 3.3. Prediction based on Molecular Dynamics Simulation Techniques

In road materials, molecular dynamics simulation techniques have become an excellent way to reveal the mechanisms of material behavior. Fluorescence microscopy and AFM techniques can only observe the modifier′s dispersion in the matrix bitumen and the structural changes following the action of the modifier on the bitumen. However, they cannot relate the structural changes to the properties of the bitumen [116]. The infrared spectroscopy technique identifies the components resulting from the reaction between the modifier and the bitumen by analyzing characteristic absorption peaks, which can be analyzed qualitatively. However, these methods can only be used to study in detail the reasons for the influence of modifiers on the properties of bitumen [117,118]. Molecular dynamics simulations have been introduced to reveal the effects of modifiers on asphalt properties.

**Figure 9:**
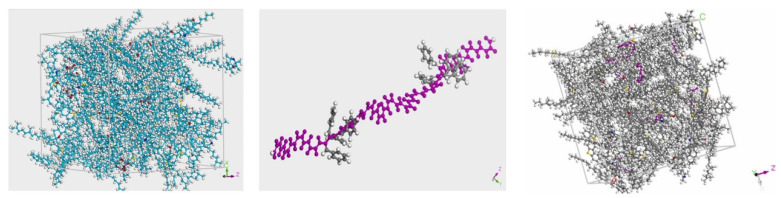
Asphalt molecular model, SBS molecular model, and SBS-modified asphalt mixture model [116].

The principle of the molecular dynamic simulation technique is to model molecules at the atomic level and reveal physical and chemical properties via computer simulation of molecular structure and motion [119,120,121]. Several scholars have studied the compatibility of bitumen and modifiers through molecular dynamics simulations. Ding et al. analyzed the effect of SBS on the agglomeration behavior of bitumen molecules and also investigated the compatibility mechanism between SBS modifiers and bitumen [122,123,124]. Liuet al. [125] established a molecular model of bitumen and SBS with different block ratios at the microscopic level. The asphalt–SBS interaction layer system was installed, and the diffusion and adhesion effects of SBS with varying proportions of the block were evaluated by calculating the immersion depth, diffusion coefficient, and adhesion work of SBS in asphalt. Luo established molecular models of the direct-to-plant SBS modifier, asphalt, and minerals and predicted the basic properties of composite-modified asphalt and the interaction between composite-modified asphalt and minerals. At the macro level, the modification effect of the modifier was verified through basic performance tests on asphalt and asphalt mixtures [27]. Su used Materials Studio software to construct a molecular model of asphalt, a molecular model of SBS, and a model of the SBS-modified asphalt mixture (as shown in Figure 9) to study the co-blended system of SBS and matrix asphalt, providing insight into the compatibility of SBS with asphalt [116]. Luo modeled the interaction between asphalt components and SBS molecules during swelling [126]. The effect of each element on the swelling pattern was discussed. Some scholars have also investigated the self-healing and aging of bitumen using molecular dynamics simulations [127,128,129,130,131]. Hu and Luo [132,133] investigated the self-healing and interfacial properties of mastic powder-modified bitumen using thermodynamic parameters. Xu found a molecular model of aging asphalt by introducing functional groups into the matrix and studied the oxidative aging effect of asphalt binders [134]. Meng established three asphalt aging models (unaged, short-term aged, and long-term aged) to study diffusion patterns and mechanisms under different influencing factors [135].

In conclusion, molecular dynamics simulations have become a valuable and essential tool in studying asphalt. Molecular dynamics can relate the microscopic mechanism of the material to the macroscopic mechanical properties, build predictive models at the microscopic level, and test the performance of the mix at the visible level by testing the Marshall stability, residual stability, freeze–thaw splitting strength ratio, dynamic stability in rutting experiments, and low-temperature bending damage strain to verify the accuracy of the predictions.

## 4. Construction Technology of Direct-to-Plant SBS-Modified Asphalt

**Figure 10:**
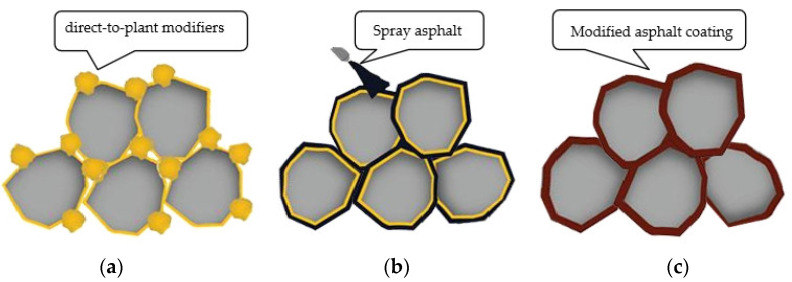
Modification process of direct-to-plant SBS modifier, (**a**) represents the direct casting SBS modifier and dry mixing of aggregates, (**b**) represents the wet mixing of sprayed asphalt, and (**c**) represents the formation of a modified asphalt coating.

Preparing direct-to-plant SBS-modified asphalt mixes is to pre-mix the direct-to-plant SBS modifier with a coarse aggregate in a dry mix, mix well, and then add asphalt, fine aggregate, and mineral powder for wet mixing, as shown in Figure 10, (a) represents the direct casting SBS modifier and dry mixing of aggregates, (b) represents the wet mixing of sprayed asphalt, and (c) represents the formation of a modified asphalt coating. Compared to the traditional damp production of SBS-modified bitumen, direct-to-plant SBS modification has more advanced technical principles and performance advantages, paving the mix at the optimal point of change of the modifier performance to maximize the traditional wet modification in the decay process of implementation. Direct-to-plant SBS mixes a variety of road performance factors as an explicit criterion, while in the conventional wet change of the supplier-modified asphalt’s indicators of control mode, there are significant defects; in the use of the process of quality management, direct-to-plant SBS is simple, high quality, and transparent. The direct-to-plant SBS modification process eliminates the modified asphalt plant, and the modified energy consumption is small.

**Figure 11:**
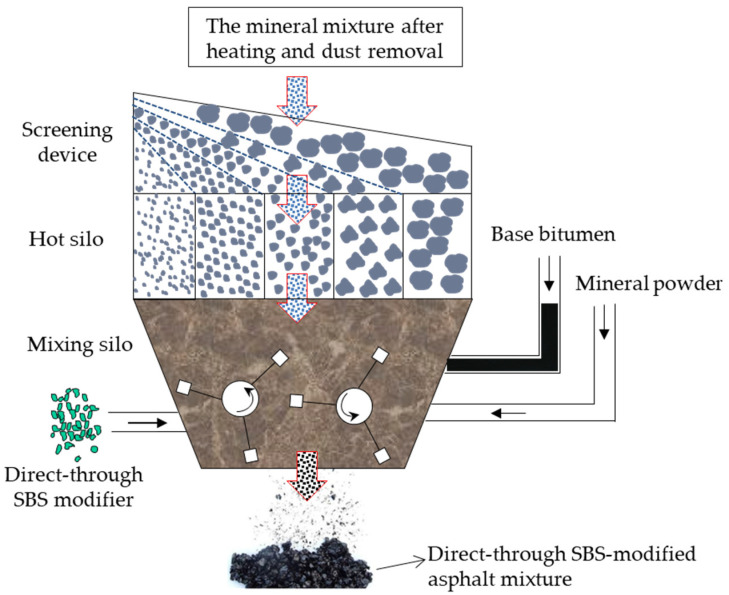
Production diagram of direct-to-plant SBS-modified asphalt mixture.

The direct-to-plant SBS-modified bitumen construction process can be divided into four stages: The design stage, raw material preparation stage, mix ratio design stage, and site construction stage:

(1) Design stage: This is in accordance with the four aspects of the construction area: Road grade, climatic conditions, traffic conditions, and road-surface type. It is combined with site engineering experience to design a reasonable construction plan.
(2) The selection stage of raw materials: The selection of raw materials includes the direct-to-plant SBS modifier (for technical specifications, see Table 1 below), matrix asphalt, aggregates, etc., in which the direct-to-plant SBS-modified asphalt mixture should choose a matrix asphalt with high compatibility after being mixed with the direct-to-plant SBS modifier. The SARA content of the asphalt will directly affect the performance of the modified asphalt. When selecting the matrix asphalt, compatibility with the modifier should be checked [136,137,138].

(3) Design phase: The direct-to-plant SBS-modified asphalt mix design should be based on the mix type. The oil-to-stone ratio should be the percentage of the total mass of both the base asphalt and the direct-to-plant SBS modifier to the group of the aggregate. The design phase is divided into three stages: Target mix design, production mix design, and production mix design verification.
(4) On-site construction phase: The direct-to-plant of SBS modifier and matrix asphalt, aggregate mixing, and placement can be used manually or mechanically. When mixing with the manual order of the direct-to-plant SBS modifier, the direct-to-plant SBS modifier should be divided into small portions in advance according to the designated dosage and manually placed directly into the floor mixing cylinder for mixing. After the direct-drop SBS modifier is placed into the mixing cylinder, it should be mixed dry with the hot aggregate for 5–10 s and then placed into the asphalt and mineral powder with the use of mechanical input mixing and the direct-to-plant SBS modifier, as shown in Figure 11. We set the drop time and drop volume each time the drop time is less than 10 s and the mass error of the drop volume is within 3%. The SBS-modified asphalt mix should be uniform, with no white material, coarse and fine material separation, lumping, or other phenomena.

Because the rapid melt crosslinking mechanism and behavioral characteristics of direct-to-plant SBS modifiers need to be more explicit, there is room for further optimization of the direct-to-plant SBS-modified bitumen construction process. In addition, considering the effect of time, temperature, development time, and other processing parameters on the performance of SBS-modified asphalt, the selection of appropriate parameters can improve the dispersion of modifiers in asphalt and the compatibility, thereby there is a need to strictly control the processing parameters in construction.

## 5. Conclusions

This paper systematically reviews the current status and achievements of domestic and international research on direct-to-plant SBS-modified asphalt, analyzes the material composition and mechanism of action of common direct-to-plant SBS modifiers, summarizes the evaluation method of the modification effect, and elaborates on the construction process with the following relevant conclusions:

(1) This summary divides the joint direct-to-plant SBS modifiers into four main categories, depending on the mechanism of action. All four direct-to-plant SBS modifiers are based on small-particle-size SBS as the main material composition, supplemented by additives. Direct-to-plant SBS modifiers based on the principle of rapid solubility and intra-molecular lubrication improve the melting efficiency of the modifier and its compatibility with the bitumen by adding additives. Direct-to-plant SBS modifiers based on the no-pelletizing principle use burr generators to form burrs of varying lengths on the surface of SBS pellets, increasing the contact area and eliminating the need for pelletizing and improving production efficiency. Direct-to-plant SBS modifiers based on the vulcanization reaction change the asphalt components by adding sulfur to enhance the stability of the asphalt. Currently, the joint direct-to-plant SBS modifier on the market meets the standard of immediate injection construction; there is no high-performance direct-to-plant SBS modifier.
(2) In the evaluation of the modification effect of direct-to-plant SBS-modified asphalt, the dispersibility of the direct-to-plant SBS modifier was determined by observing the particle residue after dry mixing; the macroscopic indicators of modified asphalt and modified asphalt mixture were used to determine the cross-linking effect between the direct-to-plant SBS modifier and asphalt; on the microscopic level, the modification mechanism and modification effect of the wet SBS modifier were evaluated by fluorescence microscopy, AFM, and molecular dynamics simulation techniques. At the microscopic level, wet SBS modifiers′ mechanism and modification effect are evaluated by fluorescence microscopy, the AFM technique, and molecular dynamics simulation. There is a lack of proprietary methods for assessing the modification effect of direct-to-plant SBS-modified bitumen. The existing evaluation methods are based on wet modifications, which are detached from the actual conditions of use.
(3) When comparing direct-to-planting SBS-modified production to the traditional wet production of SBS-modified asphalt, the construction process is simple, eliminating the production of modified asphalt to prevent the production process of modified asphalt environmental pollution, as well as energy consumption problems, and the high-temperature stability and water stability are superior. However, the direct-to-plant SBS-modified bitumen construction process uses traditional SBS-modified bitumen process construction and has not formed an exclusive construction process. Under the premise that the above problems exist, there is room for further optimization and enhancement of the mixing process specified in the Technical Guide for Highway Dry SBS-Modified Asphalt Pavements (T/CHTS 20003–2018).

It is recommended to strengthen the direct-to-plant SBS-modified asphalt-related performance improvement, such as the direct-to-plant-altered warm asphalt mix and flame-retardant effect. Simplifying the production process to solve the problem of asphalt modifier segregation at the same time so that the asphalt mixture construction temperature is reduced and has flame-retardant properties is a significant trend in the future development of direct-to-plant SBS-modified asphalt. In addition, the research and development of high-performance direct-to-plant SBS modifiers and complete sets of production technology for different regions and working conditions should be targeted to strengthen the accumulation of practical experience to form a complete theoretical system.

## Figures and Tables

**Table 1:** Technical requirements for direct-to-plant SBS modifier [20].

Test Items	Unit	Technical Requirement
SBS content	%	≥50
ash content	%	≤1
Melt index	g/10 min	≥2
Dry-mixed dispersibility	-	No particle residue

## Data Availability

Not applicable.
